# Supplementary material for: Validation of the Internal Coherence Scale (ICS) in Healthy Geriatric Individuals and Patients Suffering from Diabetes Mellitus Type 2 and Cancer
Source: Geriatrics (Basel). 2024 May 14;9(3):63. doi: 10.3390/geriatrics9030063 (PMC11130969; doi:10.3390/geriatrics9030063)
Supplement: Supplementary file 1 [file geriatrics-09-00063-s001.zip › Table S1_demographics_130524.pdf]

Table S1. Demographic Characteristics of the geriatric sample.

	**Groups**						***p*-value**
**Variables**	**Oncology Group** (O) n = 31		**Diabetes Group** (D) n =22		**Healthy Control** (C) n = 51		
	**M**	**SD**	**M**	**SD**	**M**	**SD**	
**Age (in years)**	80.61	6.68	80.32	4.90	81.16	6.98	.839
range	71-94		72-87		70-96		
	**N**	**in %**	**N**	**in %**	**N**	**in %**	
**Gender**							.316
Female	19	61.29	11	50.00	35	68.63	
Male	12	38.71	11	50.00	16	31.37	
**Living Situation**<br>with partner	21	67.74	12	54.55	20	39.22	**.012****⸸<br>**.040***; O↔H⸸
**Children (yes)**	27	87.10	22	100	38	74.51	**.009****⸸<br>**.021***; D↔H ⸸
**Family status**							.278
Single	3	9.68	0	0	5	9.80	
Married	18	58.06	11	50.00	20	39.22	
Divorced	3	9.68	2	9.09	2	3.92	
Widowed	7	22.58	9	40.90	23	45.10	
**Education**							.235
Junior High School	8	25.81	13	59,09	15	29.41	
Secondary School	10	32.26	4	18.18	15	29.41	
A-Level	12	38.71	5	22.73	16	31.73	
Other	1	3.23	0	0	3	5.88	
Without graduation	0	0	0	0	2	3.92	
**Higher Education**							.257
Apprenticeship	10	32.26	11	50.00	17	33.33	
Vocational school	3	9.68	0	0	8	15.69	
University of Applies Science	5	16.13	4	18.18	7	13.73	
University	10	32.36	4	18.18	14	27.45	
Others	2	6.45	0	0	4	7.84	
No degree	1	3.23	3	13.64	1	1.96	

$p < 0.05$*; $p < 0.01$** in bold; Chi-Square-Tests (*p*-Value) for gender, living situation, children, family status, education and work ⸸ significant group comparison for O↔H and D↔H; Kruskal-Wallis-Test (*p*-Value).
