# Supplementary material for: Validation of the Internal Coherence Scale (ICS) in Healthy Geriatric Individuals and Patients Suffering from Diabetes Mellitus Type 2 and Cancer
Source: Geriatrics (Basel). 2024 May 14;9(3):63. doi: 10.3390/geriatrics9030063 (PMC11130969; doi:10.3390/geriatrics9030063)
Supplement: Supplementary file 1 [file geriatrics-09-00063-s001.zip › Table S2_PCA_130524.pdf]

Table S2. Items of the ICS with factor loading, factor assignment and explained variance, alpha if item deleted value and item-total correlation. Cronbach's alpha mean and standard deviation are presented for the sum score, and subscales.

**ICS Items (10)** n = 104 patients	**Factor loadings**				**Item-Scale Statistics**		
**Items**	**1**		**2**		**Item total correlation**	**Means (SD) t1**	**Alpha if item deleted**
Subscale 1: Internal Coherence and Resilience	**70-96 years**	**30-83 years ***			ICS-elderly version: 70-96 years		
5. Confidence during day	**.75**	**.86**			.635	**4.4 (0.78)**	.677
1. Moments of comfort	**.65**	**.72**			.502	**4.2 (0.86)**	.669
10. Being on the right path	**.59**	**.71**			.334	**4.4 (0.78)**	.697
9. Feeling secure	**.60**	**.84**			.353	**4.3 (0.90)**	.694
4. Health condition	**.57**	**.64**			.459	**3.8 (0.85)**	.677
7. Ideas to solve problems	**.67**	**.73**			.500	**4.3 (1.10)**	.665
8. Feeling of coherence	**.48**	**.77**			.278	**4.1 (1.00)**	.708
6. Feeling of courage	**.46**	**.58**			.273	**4.6 (0.91)**	.707
Subscale 2: Thermo Coherence			**70-96 years**	**30-83 years**			
2. I was cold			**.86**	**.90**	.184	4.5 (0.92)	.722
3. I felt warm			**.81**	**.89**	.267	4.4 (0.87)	.707
**Mean Sum Score (SD)**						42.8 (4.76)	
**Cronbach's Alpha = .72**	.75		.73			**Test-retest rtt = .53**	
