# Supplementary material for: Validation of the Internal Coherence Scale (ICS) in Healthy Geriatric Individuals and Patients Suffering from Diabetes Mellitus Type 2 and Cancer
Source: Geriatrics (Basel). 2024 May 14;9(3):63. doi: 10.3390/geriatrics9030063 (PMC11130969; doi:10.3390/geriatrics9030063)
Supplement: Supplementary file 1 [file geriatrics-09-00063-s001.zip › Table S3_correlations_13524.pdf]

Table S3. Partial Spearman rank correlations of the geriatric ICS scores and subscales, Trait aR, SOC, SF-12, KPI, GDS stratified by diagnostic groups.

	1	2	3	4	5	6	7	8	9
1 ICS SumScore	1	**.943****	**.470****	**.455****	**.493****	**.399****	**.542****	**.221***	**-.348****
2 ICS Coherence & Resilience	**.943****	1	.150	**.392****	**.474***	**.334****	**.564****	**-.237****	**-.373****
3 ICS Thermo Coherence	**.470****	.150	1	**.331****	**.206***	**.299****	.113	.028	-.044
4 Trait aR	**.455****	**.392****	**.331****	1	**.374****	**.466****	**.266****	**.315****	**-.330****
5 SOC	**.493****	**.474***	**.206***	**.374****	1	.177	**.460****	.077	**-.270****
6 SF-12 physical	**.399****	**.334****	**.299****	**.466****	.177	1	-.034	**.473****	-.284
7 SF-12 psychological	**.542***	**.564****	.113	**.266****	**.460****	-.034	1	.103	-.213
8 KPI	**.221***	**-.237****	.028	**.315****	.077	**.473****	.103	1	**-.391****
9 GDS	**-.348****	**-.373****	-.044	**-330****	**-.270****	-.284**	-.213*	**-.391****	1

* p <.05, ** p <.001 Partial Spearman rho, 2-tailed in bold
