# Supplementary material for: Validation of the Internal Coherence Scale (ICS) in Healthy Geriatric Individuals and Patients Suffering from Diabetes Mellitus Type 2 and Cancer
Source: Geriatrics (Basel). 2024 May 14;9(3):63. doi: 10.3390/geriatrics9030063 (PMC11130969; doi:10.3390/geriatrics9030063)
Supplement: Supplementary file 1 [file geriatrics-09-00063-s001.zip › Table S4_ART_130524.pdf]

Table S4. $p < 0.05$*; $p < 0.01$** in bold; Aligned Rank Test (*p*-Value) for Healthy Control Group, Longterm Oncology (LongONCO), Shortterm Oncology (ShortONCO), Longterm Diabetes (LongDIAB) and Shortterm Diabetes (ShortDIAB) ⸸ significant group comparison.

**ICS Mean (SD) n = 104**	Healthy Control Group (n = 51)	LongONCO (> 5 years) (n = 19)	ShortONCO (<= 5 years) (n = 10)	LongDIAB (> 5 years) (n = 17)	ShortDIAB (> 5 years) (n = 5)	**Aligned Rank Test** *p-Values*
ICS sum score	43.2 (3.7)	44.3 (5.7)	42.5 (4.1)	41.4 (5.3)	42.4 (6.6)	**.03***; HC↔LongONCO ⸸<br>**.01****LongONCO↔LongDIAB⸸
ICS coherence & resilience	34.4 (3.4)	34.8 (4.9)	34.0 (3.0)	32.6 (4.6)	33.0 (5.8)	**<.01*** LongONCO↔LongDIAB⸸<br>**.03*** **ShortDIAB**↔LongDIAB⸸<br>**.04*** LongONCO↔HC⸸
ICS thermo coherence	8.8 (1.7)	9.4 (1.1)	8.5 (2.2)	8.8 (1.4)	9.4 (0.9)	-
